# Through Parents' Eyes: Perspectives on Child Development and Family Experience During ESDM Intervention

**DOI:** 10.1002/aur.70318

**Published:** 2026-07-25

**Authors:** Kenza Latrèche, Fiona Journal, Michel Godel, Martina Franchini, Nada Kojovic, David Cimasoni, Marcin Czajkowski, Lucie Delieutraz, Chloé Laubu, Marie Schaer

**Affiliations:** ^1^ Department of Psychiatry Faculty of Medicine, University of Geneva Geneva Switzerland; ^2^ Fondation Pôle Autisme Geneva Switzerland; ^3^ Division of Adult Psychiatry, Department of Psychiatry University Hospitals of Geneva Geneva Switzerland; ^4^ Faculty of Psychology and Educational Sciences University of Geneva Geneva Switzerland; ^5^ Parent Collaborator and Community Contributor Geneva Switzerland

**Keywords:** autism spectrum disorder, children, early intervention, Early Start Denver Model, parental perspectives, parenting skills, well‐being

## Abstract

Early intensive intervention for autistic children aims to support developmental progress in social and communication domains. Although outcomes are typically assessed using standardized measures of child development, parental perspectives during early intervention remain largely understudied. Here, we examined parental perceptions after 6 months of intensive Early Start Denver Model (ESDM) intervention. Participants were parents of 104 autistic children aged 1.3–3.5 years enrolled in an individualized therapist‐delivered ESDM intervention following diagnosis confirmation. Child development was assessed at entry (baseline) and 6 months later (follow‐up). At follow‐up, parents completed an 18‐item questionnaire capturing their perceptions of child progress across twelve domains as well as changes in parenting skills, personal well‐being, and family life. Descriptive analyses, item‐level correlations, and principal component analysis (PCA) were conducted to identify underlying components. Parents generally indicated improvements across all domains, with aggravation rarely reported. PCA revealed three intercorrelated components (Social Communication, Nonverbal Social Engagement, and Positive Parental Experience), each showing distinct associations with child or parent measures. Parental perceptions of child development were strongly correlated with standardized developmental assessments, particularly in social communication. While parents also reported enhanced parenting skills and positive personal and familial impacts, these experiences were not associated with child developmental gains but with parental education and the number of siblings in the household. These findings underscore the importance of integrating parental perspectives alongside clinical assessments when evaluating early intervention outcomes. Future research should investigate how parental perceptions evolve over longer periods and how they may inform family‐centered care and post‐intervention support.

## 1 Introduction

Autism spectrum disorder (henceforth, autism) is a neurodevelopmental condition characterized by challenges in social communication and interactions, alongside restricted and repetitive behaviors and interests (American Psychiatric Association 2013). Early diagnosis is crucial as timely interventions can leverage brain plasticity to support developmental gains and autonomy (Dawson 2008; Harris and Handleman 2000; Sullivan et al. 2014). Naturalistic Developmental Behavioral Interventions (NDBIs) are evidence‐based approaches integrating principles of operant learning and developmental science to promote skill acquisition in naturalistic contexts (Schreibman et al. 2015; Vivanti and Stahmer 2021). Among NDBIs, the Early Start Denver Model (ESDM) is a well‐recognized, empirically validated intervention for children aged 12–48 months, targeting core domains such as language and social engagement (Vivanti and Stahmer 2021; Dawson et al. 2010; Rogers et al. 2012). ESDM is typically delivered intensively in a one‐to‐one setting, with 15–25 h of weekly therapist‐led sessions, making parents' active involvement essential to their child's participation and learning (Dawson et al. 2010; Godel et al. 2022; Vivanti et al. 2016; Rogers et al. 2019).

Given this central role, parents' experiences throughout diagnostic and intervention processes are critical but remain relatively underexplored. The diagnostic process is a well‐recognized source of stress (Rabba et al. 2019; DesChamps et al. 2020), often requiring coordination of multiple appointments, time away from work, and navigation of financial, linguistic, or cultural barriers (Aylward et al. 2021; Howes et al. 2021; Malik‐Soni et al. 2022). After diagnosis, parents frequently face additional responsibilities, including accessing qualified healthcare providers and advocating for their child (Boshoff et al. 2018; Bonis 2016; Bent et al. 2023; Mendez et al. 2024). During this time, parents of autistic children frequently experience overwhelm, fear of the unknown, and lack of control (Rabba et al. 2019).

Early intervention, while requiring substantial investment in time and financial resources, can offer parents a pathway to regain agency and hope. In ESDM, parents learn to implement intervention strategies within daily routines through coaching sessions. This collaborative role can produce both positive and challenging experiences (Bent et al. 2023; Edwards 2014). Parents may feel increased empowerment and self‐efficacy in supporting their child, while simultaneously experiencing pressure to consistently apply strategies and maximize learning opportunities. Associations between parental efficacy, stress, and mental health further highlight the importance of capturing parental experiences during early intervention (Brennan et al. 2024; Gentile et al. 2022).

Although parents play central roles in early intervention, research has predominantly focused on child outcomes (e.g., language, autism severity (Dawson et al. 2010; Rogers et al. 2012; Godel et al. 2022; Warren et al. 2011)). Studies examining parental outcomes (i.e., the effects of ESDM on parents) mostly involved low‐intensity parent‐implemented programs (e.g., P‐ESDM), showing benefits in feasibility (i.e., achieving intervention goals), fidelity (i.e., correct and consistent implementation), and satisfaction (Carlsson et al. 2024; Rogers et al. 2022; Van Noorden et al. 2022). Parental outcomes in intensive one‐to‐one therapist‐delivered interventions remain underexplored. Some studies reported reduced parenting stress but findings are constrained by low‐intensity formats, group settings, and small samples (Bent et al. 2023; Feng et al. 2025; Ogilvie and McCrudden 2017; Levato et al. 2025). Understanding parents' perspectives in intensive ESDM is critical, as their engagement, perceptions of child progress, and well‐being can influence intervention effectiveness and child outcomes (Bent et al. 2023; Estes et al. 2019; Vivanti et al. 2014; Karst and Van Hecke 2012).

To address these gaps, the present study examined parental perceptions during an intensive individualized ESDM intervention over the first six months, a transition period during which families must adapt to a demanding schedule, and one that is critical for predicting children's outcomes (Godel et al. 2022). Our sample included parents of 104 autistic children, aged 1.3–3.5 years. We developed a parent‐report questionnaire to capture perceptions of both child progress and parental experience. This study had three objectives: (1) to describe parents' perceptions of their child's progress, their own parenting skills, and personal and familial well‐being; (2) to identify the underlying dimensions of these perceptions using principal component analysis; (3) to examine how these dimensions relate to objective child clinical measures and parental characteristics. We hypothesized that after 6 months of intensive ESDM, parents would generally report positive perceptions of their child's development, as well as improvements in their own skills. We also hypothesized that parental perceptions would be more positive when children demonstrated greater objective developmental progress.

## 2 Methods

### 2.1 Participants

Prior to any study inclusion, all children were referred for an autism diagnostic evaluation in the cantonal center for autism in Geneva. All children were assessed by trained psychologists and psychiatrists using gold‐standard diagnostic tools (ADOS, ADI‐R) (Lord et al. 1994, 2012). The Mullen Scales of Early Learning (MSEL) (Mullen 1995) were also administered to evaluate the children's developmental level. Following confirmation of an autism diagnosis, parents were offered the opportunity, subject to availability, to enroll their child in a two‐year intensive early intervention program based on the Early Start Denver Model (ESDM). Families who accepted enrollment in the ESDM intervention were subsequently invited to participate in the Geneva Autism Cohort, a longitudinal cohort designed to follow children's developmental trajectories throughout the intervention period.

The Geneva Autism Cohort longitudinally follows young autistic children (Journal et al. 2024; Latrèche et al. 2024) and conducts assessments every 6 months over the two‐year ESDM intervention, resulting in a total of five time points. The present study focused on data collected at baseline (intervention entry) and at the six‐month follow‐up. The baseline assessment was conducted at early intervention program entry and included the ADOS‐2 (Lord et al. 2012), the MSEL (Mullen 1995), and the VABS‐II (Sparrow et al. 2005), all administered by trained psychologists. In addition, parents completed questionnaires collecting information about demographics, as well as parental depression and anxiety. The follow‐up assessment, conducted 6 months after program entry, consisted of the MSEL, the VABS‐II, and several parent‐report questionnaires, including the Parental Perceptions of Change Questionnaire, which we developed.

To be included in the present study, children were required to have a confirmed ASD diagnosis, be enrolled in the ESDM intervention program, have available baseline and 6‐month follow‐up MSEL data, and have a parent who completed the Parental Perceptions of Change Questionnaire at follow‐up. For one child, baseline MSEL data were unavailable; in this case, the PEP‐3 (Schopler et al. 2005) was used instead. In total, the sample included 104 autistic children.

Sociodemographic characteristics are presented in Table 1 and sample characteristics are described in Table 2.

**TABLE 1:** Sociodemographic characteristics of the parents of 104 autistic children.

*n =* 104	Proportion [%], *n*
Questionnaire respondent [*n* = 104]	
Biological mother	77.9% [*n* = 81]
Biological father	11.5% [*n* = 12]
Both biological parents	9.6% [*n* = 10]
Foster mother	1.0% [*n* = 1]
Marital status [*n* = 104]	
Married or in a relationship	91.3% [*n =* 95]
Separated or divorced	8.7% [*n =* 9]
Number of sibling(s) [*n* = 103^a^]	
None	37.9% [*n =* 39]
1	42.7% [*n =* 44]
2	17.5% [*n =* 18]
4–6	1.9% [*n =* 2]
Sibling order position of the autistic child [*n* = 103^a^]	
1st‐born child	50.5% [*n =* 52]
2nd‐born child	34.0% [*n =* 35]
3rd‐born child	13.6% [*n =* 14]
4th‐6th born child	1.9% [*n =* 2]
Parental education [*n* = 88]	
End of primary school (6th grade)	0.0% [*n =* 0]
Lower secondary school (9th grade)	4.5% [*n =* 4]
Partial secondary education	2.3% [*n =* 2]
Completed secondary education	29.5% [*n =* 26]
Partial university studies	8.0% [*n* = 7]
Completed university studies	40.9% [*n =* 36]
Postgraduate/doctoral studies	14.8% [*n =* 13]
Annual household income [*n* = 92]	
< 15,000 to 60,000 CHF (< 16,500 to 66,000 USD)	34.8% [*n =* 32]
60,000 to 140,000 CHF (66,000 to 154,000 USD)	43.5% [*n =* 40]
140,000 to > 240,000 CHF (154,000 to > 264,000 USD)	21.7% [*n =* 20]

	*M*	SD
Age at birth		
Mother	32.69 [*n* = 99]	6.16
Father	37.90 [*n* = 97]	6.11

*Note:* We considered half‐siblings only if they lived in the same household as the autistic child.

Abbreviations: CHF, Swiss Franc; USD, United States Dollar.

^a^ One child who lived in a foster family without any biological siblings was excluded from these analyses.

**TABLE 2:** Sample characteristics at the baseline and follow‐up assessments.

*n =* 104	Baseline assessment intervention start		Follow‐up assessment 6 months of intervention		*p*
	*M*	SD	*M*	SD	
Age (months)	29.72	5.19	36.14	5.25	
MSEL total DQ	60.69	16.59	70.27	20.89	< 0.001
MSEL verbal DQ	46.16	20.42	59.87	26.14	< 0.001
MSEL nonverbal DQ	75.23	16.55	80.66	18.08	< 0.001
VABS‐II adaptive behavior standard score	78.85 (*n* = 103)	9.19	81.33 (*n* = 102)	10.96	< 0.001
ADOS‐2 total severity score	8.00	1.88			
HADS parental Anxiety total score	7.32 (*n* = 93)	4.16	6.54 (*n* = 100)	3.62	0.036
HADS parental depression total score	6.25 (*n* = 93)	3.94	5.96 (*n* = 100)	4.10	0.888

*Note:* The ADOS‐2 was administered only at baseline. The *p*‐values result from Wilcoxon‐signed rank tests comparing measurements at intervention start and after 6 months of intervention.

Abbreviations: ADOS‐2, Autism Diagnostic Observation Schedule, 2nd edition; DQ, developmental quotient; HADS, Hospital Anxiety and Depression Scale; *M*, mean; MSEL, Mullen Scales of Early Learning; SD, standard deviation; VABS‐II, Vineland Adaptive Behavior Scales, 2nd edition.

### 2.2 ESDM Intervention

The early and intensive intervention program, based on the Early Start Denver Model (ESDM), takes place in the Centre d'Intervention Précoce en Autisme (CIPA) centers in Geneva. The CIPAs are run either by a private foundation (Fondation Pôle Autisme, *N* = 82), or by the State (Office Médico‐Pédagogique *N* = 22). The ESDM intervention is implemented in an individualized setting (i.e., one therapist for one child). The intensity of the intervention averaged 18.6 h per week (range: 15–20 h) (for more details, see (Godel et al. 2022)). In addition, all parents participating in the program received one weekly hour of coaching sessions from the center's psychologists for the entire intervention period, based on the Parent‐implemented Early Start Denver Model (P‐ESDM). P‐ESDM aims to strengthen parents' existing skills and develop new abilities to incorporate learning opportunities for their child into daily routines (Vismara and Rogers 2018).

### 2.3 Measures

#### 2.3.1 Parental Perceptions of Change Questionnaire

We designed a questionnaire to examine parents' experiences during the first 6 months of early intervention. The questionnaire comprised 18 items, divided into two categories: child‐focused (12 items) and parent‐focused (6 items). The child‐focused items examined perceptions regarding their child's progress across 12 domains related to verbal and nonverbal communication, daily living, social functioning (e.g., autonomy in daily acts, play, social reciprocity with parents and children), and autistic characteristics (e.g., restricted interests, repetitive behavior). Each question used a 7‐point Likert‐scale for rating their child's evolution from “Greatly worsened” to “Greatly improved”. The responses were coded in a −3 to 3 scale for analysis, where 0 represents no change, facilitating interpretation of change direction.

The parent‐focused items assessed the perceived impact of their child's evolution over the last 6 months. Three items targeted parenting skills (i.e., understanding their child's needs, connecting and communicating with their child, managing daily challenging situations), and three items addressed the impact on personal, familial, and extra‐familial well‐being. Each question used a 4‐point Likert‐scale (i.e., “No”, “Yes, a little bit”, “Yes, a lot”, “Yes, enormously”), with responses coded to a 0–3 scale, where 0 represents no impact, improving interpretability of impact magnitude.

The 18 items were selected based on clinical relevance, alignment with the objectives of the ESDM, and our clinical experience working with this population. One questionnaire per child was provided, and parents had the choice to complete it individually or together. To examine the underlying structure of the questionnaire, we conducted a principal component analysis (PCA) on item responses. The questionnaire is available in the Supporting Information.

#### 2.3.2 Developmental Level

We evaluated the baseline developmental level of children and their outcome 6 months later (follow‐up assessment) with the MSEL (Mullen 1995), a standardized developmental assessment evaluating five domains: Gross Motor (GM) and Fine Motor (FM) skills, Visual Reception (VR), Receptive Language (RL), and Expressive Language (EL). To describe the developmental level of our sample, we computed developmental quotients (DQ) by dividing developmental age equivalent score by the chronological age and multiplying by 100 (Godel et al. 2022; Lord et al. 2006). We computed the Total DQ (combining FM, VR, RL, and EL age equivalent scores), the Verbal DQ (average of RL and EL age equivalents), and the Nonverbal DQ (average of VR and FM age equivalents).

To assess developmental change between the baseline assessment and the follow‐up assessment, we calculated the Total, Verbal, and Nonverbal rates of developmental change as demonstrated below:

$$\text{Rate of developmental change}=100\times \frac{\text{Total}\;\mathrm{Age}\;\text{Equivalent}\;\mathrm{at}\;\text{Follow}\;\mathrm{up}-\text{Total}\;\mathrm{Age}\;\text{Equivalent}\;\mathrm{at}\;\text{Baseline}}{\text{Chronological}\;\mathrm{Age}\;\mathrm{at}\;\text{Follow}\;\mathrm{up}-\text{Chronological}\;\mathrm{Age}\;\mathrm{at}\;\text{Baseline}}$$

#### 2.3.3 Adaptive Functioning

The VABS‐II (Sparrow et al. 2005), administered at baseline and at follow‐up, is a semi‐structured interview with parents. The VABS‐II explores the child's adaptive functioning across four domains, namely Communication, Socialization, Daily Living skills, and Motor skills. To assess change in adaptive functioning from the baseline to the follow‐up assessment, we computed the Total, Communication, Socialization and Daily Living skills rates of change as described above.

#### 2.3.4 Parental Anxiety and Depression

Parental anxiety and depression were assessed with the parent‐report Hospital Anxiety and Depression Scale (HADS) (Zigmond and Snaith 1983). Scores of 11 or higher are considered indicative of clinically significant depression and anxiety. To examine changes in depressive and anxiety symptoms, we computed ΔHADS scores by subtracting baseline from follow‐up total scores (Δ = Follow‐up total score—Baseline total score). We received 93 completed HADS at baseline (81 by mothers, 6 by fathers, 6 by both parents) and 100 at follow‐up (88 by mothers, 3 by fathers, 9 by both parents). In total, 91 parents completed the HADS both at baseline and follow‐up.

#### 2.3.5 Demographic Questionnaire

Using a parent‐report questionnaire (Hollingshead 1975), we collected several sociodemographic variables. Parental education was categorized into seven levels: End of primary school (6th grade); Lower secondary school (9th grade); Partial secondary education; Completed secondary education; Partial university studies; Completed university studies; and Postgraduate/doctoral studies. The highest educational level attained by either parent (mother or father) was retained for the analysis.

The annual household income was grouped into three categories: less than 15,000–60,000 Swiss Francs; 60,000–140,000 Swiss Francs; and 140,000–240,000 Swiss Francs or higher. Parents reported their marital status (married or in a relationship vs. separated or divorced). Regarding siblings' information, parents reported the number of siblings or stepsiblings living in the same household, and the birth order of the autistic child among them was calculated.

### 2.4 Statistical Analysis

All analyses used nonparametric methods due to the ordinal nature and non‐normality of the questionnaire data. Analyses were performed in GraphPad Prism (Version 10.1.0) and IBM SPSS Statistics (Version 29). Significance thresholds were adjusted for multiple comparisons using Bonferroni correction as specified below.

#### 2.4.1 Descriptive Statistics

Descriptive analyses were conducted on the responses to the questionnaire items using one‐sample Wilcoxon signed rank test. Plots were made using Microsoft Excel (Version 16.102).

#### 2.4.2 Spearman Correlation Matrix of Questionnaire Items

We computed a Spearman correlation matrix among the 18 questionnaire items to examine the relationships between different domains of perceived progress and parent‐related items (e.g., parenting skills, impact on well‐being). This analysis aimed to identify clusters of potentially related items and assess the internal structure of the questionnaire. *p*‐values were Bonferroni‐corrected with an adjusted significance threshold of 0.0028 (0.05/18).

#### 2.4.3 Factor Structure of Parental Perceptions of Change Questionnaire

Following principal component analysis (PCA), we retained three factors and we applied oblimin normalized rotation to allow correlations among them (more information in Figure S4 and Table S5). Listwise case exclusion removed seven participants with incomplete data, leaving a final sample of 97 autistic children. We exported factor scores using the regression method. Factor 2 scores were inverted to facilitate interpretation.

Spearman correlation coefficients were calculated between factor scores and child clinical measures (VABS‐II Rate of Change, MSEL Rate of Change and at Baseline, ADOS Severity scores at Baseline) and parent sociodemographic and clinical measures (Annual household income, Parental Education, Marital Status, Number of siblings, Sibling order position, baseline HADS, and ΔHADS). Scatterplots were generated for significant associations and *p*‐values were Bonferroni‐corrected within each assessment subdomain (adjusted significance thresholds: 0.0125–0.0167).

### 2.5 Parent Contribution to the Study

Following data collection and statistical analyses, we contacted the local autism association (https://autisme‐ge.ch/) as well as parents previously implicated in a full‐day hackathon on how to best support parents during early intervention, to present the project and seek parents' perspectives on interpreting our results. Four parents, each with a child who completed the two‐year ESDM intervention program, participated in a two‐hour session with the first and last authors. Parents shared experiences related to the period following diagnosis, the first months of early intervention, and their overall experiences. They also offered interpretations of some results and suggested additional analyses, such as the potential influence of children's baseline developmental characteristics on parental perceptions, as well as the importance of family context, including socio‐economic status and siblings. These contributions informed the framing of the Introduction and the Discussion, including reflections on directions for future research. Following this session, all four parents expressed interest in receiving updates on additional analyses and reviewing the manuscript draft. All parents subsequently volunteered as co‐authors. Participation was voluntary and uncompensated.

### 2.6 Use of AI‐Assisted Language Tools

ChatGPT (OpenAI) was used solely for language editing; no scientific content was generated.

## 3 Results

### 3.1 Descriptive Analysis

Most families were two‐parent households (91.3%), and nearly half of the parents had a college degree (47.1%). Thirty‐nine percent earned below the Swiss median income (Table 1). Parental anxiety decreased by follow‐up (*p* = 0.036), while depression stayed stable (*p* = 0.888) (Figure S1, Table 2).

Among autistic children, 48.1% had no siblings. They showed important verbal delays at baseline but relatively preserved nonverbal skills. Six months later, they improved by approximately 10 DQ points across verbal, nonverbal, and adaptive domains (*p* < 0.001, Table 2).

### 3.2 Parental Perceptions of Change Questionnaire

**FIGURE 1:**
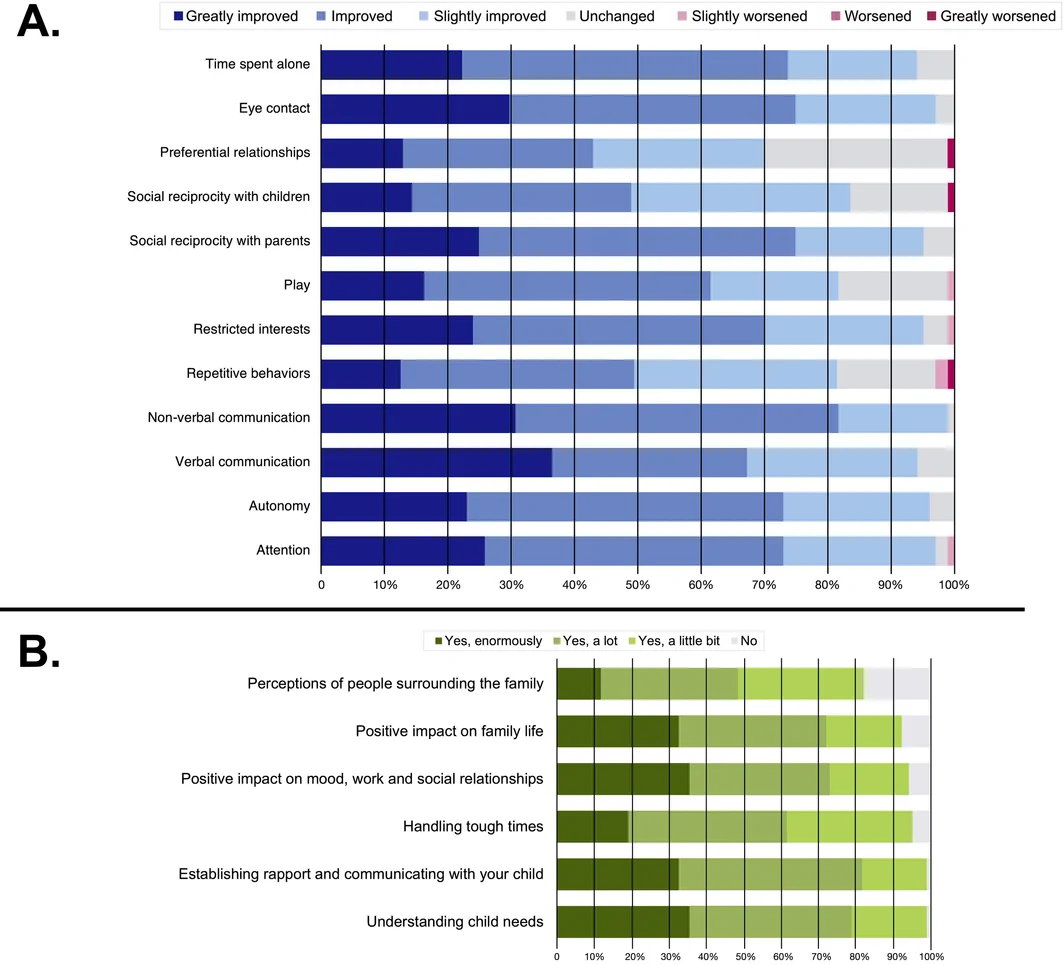
Parental responses (*N* = 104) to the 18 questionnaire items. (A) Perceived progress across 12 child‐related domains. (B) Impact of perceived progress on parents.

Of the 104 questionnaires, most were completed by mothers (78%). Overall, parents reported progress across the 12 child‐focused domains (Figure 1A), as well as improvements in their own parenting skills and positive impacts on their personal, familial, and extra‐familial lives (Figure 1B). The distribution of responses to all items is presented in Tables S1 and S2.

For each domain shown in Figure 1A, most parents reported either “Improved” or “Greatly improved” skills. The domain with the highest proportion of reported great improvements was verbal communication (36.50%). Conversely, the domain with the least reported improvements was having preferential relationships with other children, where 30.00% of the responses indicated either “Unchanged” or “Greatly worsened” skills.

Overall, negative perceptions were rare: only 0.65% of responses (8 out of 1240) indicated slightly or strongly worsened skills. Negative perceptions were observed most frequently in the Repetitive behaviors item (3 responses, 2.91%). Each of the other items (Repetitive behaviors, Social reciprocity with children, Play, Restricted interests and Attention) had one negative response each (0.96%). To better understand these negative perceptions, we identified parents who responded “Greatly worsened” or “Slightly worsened” in at least one of the 12 domains (Figure S2). These responses concerned five children in the sample. An examination of MSEL raw scores across all domains revealed no evidence of developmental regression in these five children. Compared to the 99 other children, they demonstrated similar developmental and adaptive rates of change (*p* > 0.05).

Regarding the parents of these five children, they showed a significantly greater increase in anxiety levels from baseline to follow‐up, compared to the rest of the sample, *U* = 52, *p* = 0.014 (Figure S3). The median change in HADS anxiety scores was higher in these parents (Mdn = 2.5) than in the rest of the sample (Mdn = −1.0). No significant differences were found in depression scores between the baseline and the follow‐up. Of note, these parents showed similar demographic characteristics to the rest of the sample (*p* > 0.05).

To assess the overall perceived change across the intervention, we conducted one‐sample Wilcoxon signed‐rank tests on each of the 18 questionnaire items. All items yielded statistically significant results (*p* < 0.05), indicating that perceived changes were unlikely to be due to chance. We calculated effect sizes for each item. Fifteen out of the 18 items showed a large effect size (*r* ≥ 0.8), according to Cohen's criteria, while the remaining three items demonstrated medium effect sizes (Table S3). This suggests that 6 months of intensive ESDM intervention were associated with meaningful perceived improvements across a broad range of domains.

### 3.3 Correlation Analysis of Questionnaire Items

**FIGURE 2:**
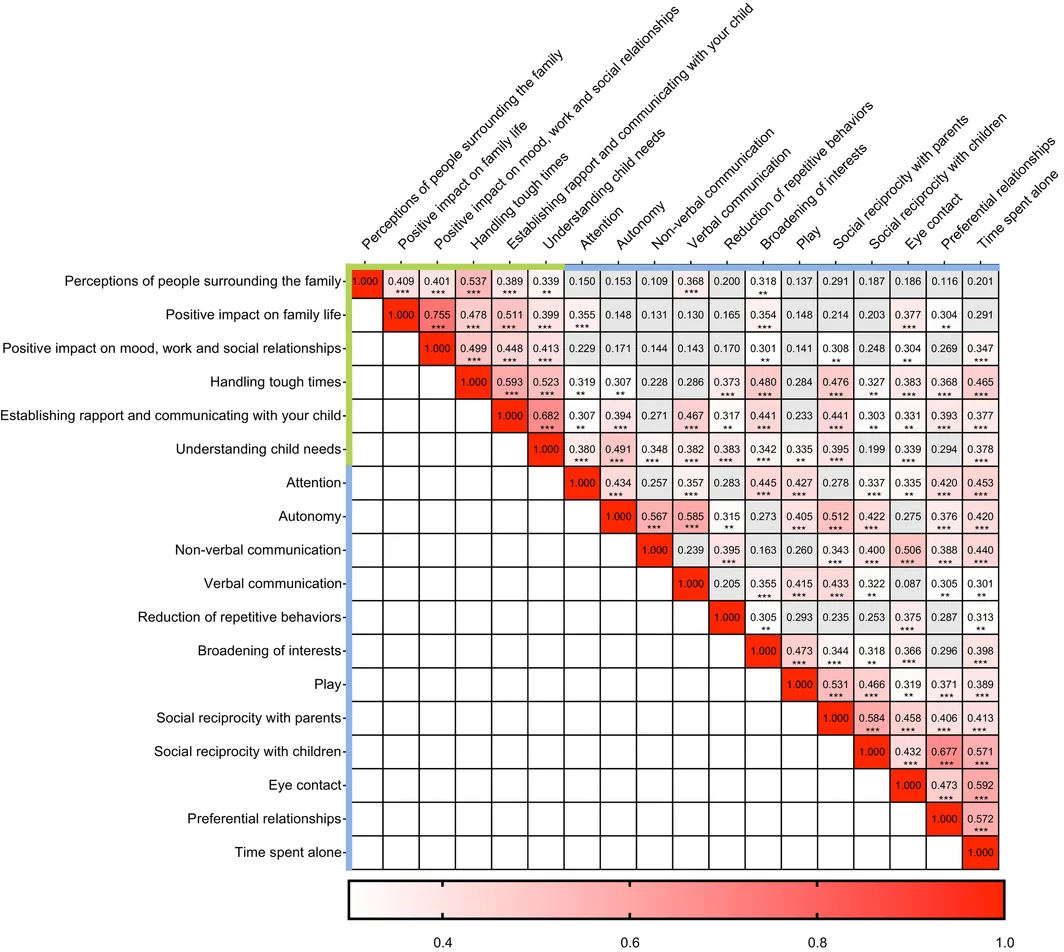
Spearman correlation matrix of the 18 questionnaire items (i.e., the 6 parent‐focused items underlined in green and the 12 child‐focused items underlined in blue). Correlations surviving Bonferroni correction are displayed in color and annotated with asterisks (*p < 0.05, **p < 0.01, ***p < 0.001). Non‐significant correlations and those not surviving Bonferroni are shown in light gray.

The Spearman correlation matrix presented in Figure 2 reveals that many items were positively and significantly correlated, with coefficients ranging from weak (*ρ* = 0.15) to strong (*ρ* > 0.75) (Dancey and Reidy 2004). *p*‐values are reported in Table S4. Correlations across the six parent‐focused items indicated moderate to strong relationships between parenting skills and perceived positive impacts of personal, familial, and extra‐familial life. The three parenting skills items were also positively correlated with several child‐related domains of progress. For instance, greater reported progress in verbal communication was significantly associated with more positive perceptions of establishing rapport and communication with their child (*p* < 0.001), as well as better understanding of their child's needs (*p* < 0.001). Some child‐related perceived progress in social reciprocity with parents, eye contact, and time spent alone, were linked to more positive impacts on personal life. Additionally, greater positive impact on family life was associated with fewer perceived restricted interests (*p* < 0.001), improved eye contact (*p* < 0.001) and increased focused attention in the child (*p* < 0.001). Perceptions of positive impact from people surrounding the family correlated with perceived improvements in verbal communication (*p* < 0.001), fewer restricted interests (*p* = 0.001), and enhanced social reciprocity with their parents (*p* = 0.003).

Moreover, the majority of the 12 child‐focused items were positively correlated, suggesting that perceived improvements in communication, social reciprocity, and behavioral domains occurred together. For instance, reported gains in verbal and nonverbal communication indicated increased involvement in social contexts (e.g., greater social reciprocity, preferential relationships, less time spent alone; *p‐*values ranged from < 0.001 to 0.002) while perceived reductions in repetitive behaviors were linked to broader interests and more varied play (*p‐*values range: from 0.002 to 0.003).

### 3.4 Factor Structure of Parental Perceptions of Change Questionnaire

PCA was conducted to explore the underlying structure of the 18 items of the Parental Perceptions of Change Questionnaire. The Kaiser‐Meyer‐Olkin (KMO) measure indicated excellent sampling adequacy for the analysis (KMO = 0.83) (Field 2013). Bartlett's test of sphericity was significant, *χ* ^2^(153) = 867.270, *p* < 0.001, demonstrating that correlations between items were sufficiently large to justify PCA. Based on the scree plot, we retained three principal factors, explaining 57.99% of the total variance (Figure S4). Component loadings and their cumulative variance explained are presented in Table S5. All items with loadings > 0.300 were retained, as each loaded highest on a single factor and contributed to the interpretability of the factor structure, consistent with PCA guidelines (Field 2013). Items were assigned to the factor on which they had the highest loading (highlighted in bold in Table S5).

The first factor accounted for 38.35% of the total variance and included the following items (ranked by factor loadings): Verbal communication, Autonomy, Play, Social reciprocity with parents, Attention, and Reduction of repetitive behaviors. We labeled this factor “Social Communication”. The second principal factor explained 11.57% of the variance and comprised: Positive impact on mood, work and social relationships, Positive impact on family life, Handling tough times, Perceptions of people surrounding the family, Establishing rapport and communicating with your child, Understanding child needs, and Broadening of interests. This factor was named: “Positive Parental Experience”. The third principal factor accounted for 8.07% of the variance and included: Preferential relationships, Social reciprocity with children, Eye contact, Time spent alone, and Nonverbal communication. We labeled this factor “Nonverbal Social Engagement”. The three factors significantly correlated with each other (*ρ* = 0.266–0.443; Table S6, Figure S5).

### 3.5 Associations Between Factor Scores and Clinical Measures

**FIGURE 3:**
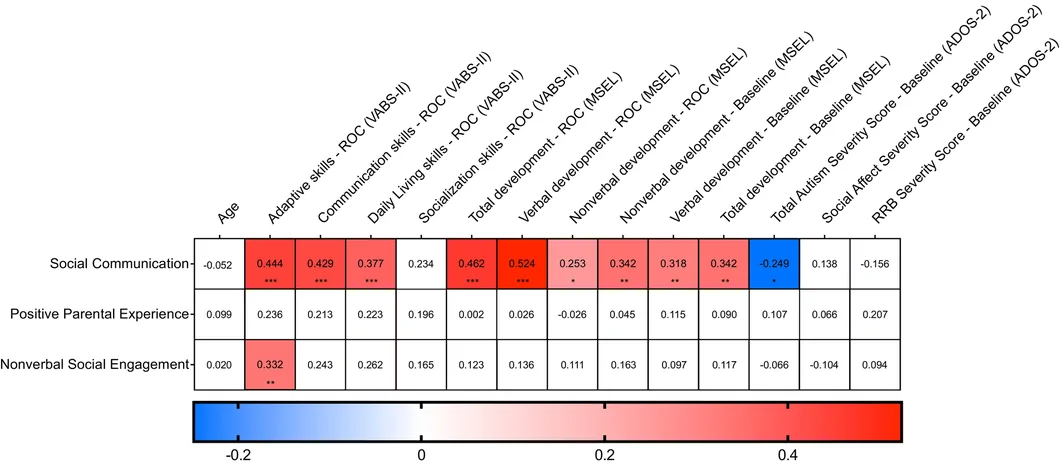
Spearman correlation matrix of three factors from the PCA and child‐related clinical variables. Correlations surviving Bonferroni correction are displayed in color and annotated with asterisks (*p < 0.05, **p < 0.01, ***p < 0.001). Non‐significant correlations and significant correlations not surviving Bonferroni are shown in white. ADOS‐2, Autism Diagnostic Observation Schedule, 2nd edition; DQ, developmental quotient; MSEL, Mullen Scales of Early Learning; ROC, Rate of Change; RRB, Restricted and Repetitive Behaviors; VABS‐II, Vineland Adaptive Behavior Scales, 2nd edition.

**FIGURE 4:**
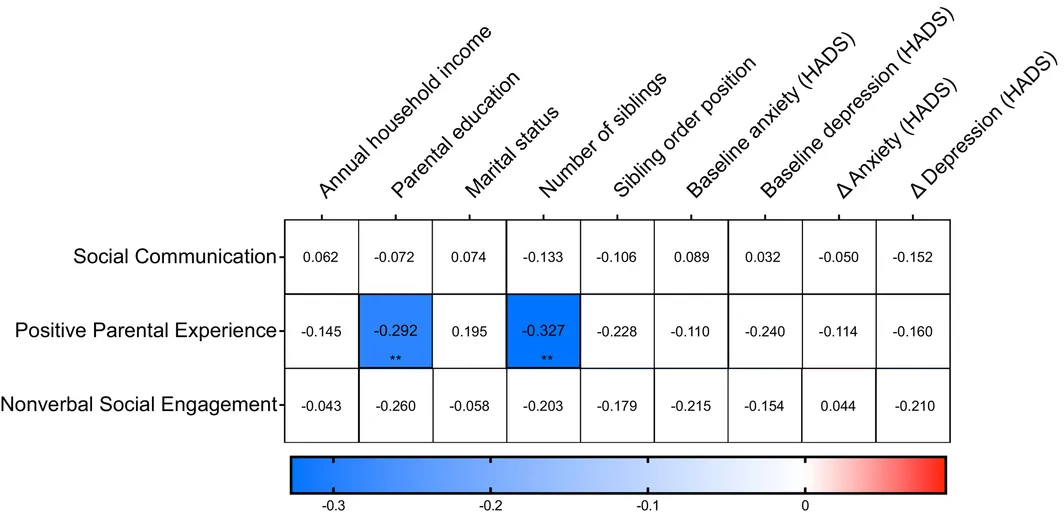
Spearman correlation matrix of three factors from the PCA and parent‐related measures. Correlations surviving Bonferroni correction are displayed in color and annotated with asterisks (**p < 0.01). Non‐significant correlations and significant correlations not surviving Bonferroni are shown in white. HADS, Hospital Anxiety and Depression Scale; Δ, HADS Anxiety Score at Follow‐up—HADS Anxiety Score at Baseline.

To examine the clinical relevance of the three PCA‐derived factors, Spearman correlations were calculated between factor scores and children‐related variables (Figure 3) as well as parent measures (Figure 4).

Regarding children‐related variables, the child's chronological age at baseline did not significantly correlate with any factor (*p* > 0.05). Social Communication was positively correlated with progress in adaptive functioning across all domains (Total, *p* < 0.001), including Communication (*p* < 0.001) and Daily Living Skills (*p* < 0.001), as well as with total (*p* < 0.001), verbal (*p* < 0.001), and nonverbal rates of change (*p* = 0.012) (Figure S6). Positive Parental Experience did not significantly correlate with any child‐related measures (*p* > 0.05). Third, Nonverbal Social Engagement was positively correlated only with VABS‐II Total adaptive progress (*p* = 0.001) (Figure S7).

Our findings regarding parent‐related variables indicate no significant correlations with Social Communication and Nonverbal Social Engagement (*p* > 0.05). This suggests that sociodemographic measures (e.g., parental education, annual household income, the number of children in the household, marital status) and parental anxiety and depression were not associated with perceived progress in their child. However, Positive Parental Experience significantly correlated with parental education (*p* = 0.007) and with the number of siblings of the autistic child (*p* = 0.001) (Figure S8). Lower parental education was linked to higher reported parenting skills and more positive well‐being, whereas having more children was linked to less positive parental experiences. Of note, the negative correlation between Positive Parental Experience and position of the autistic child among their siblings was significant before Bonferroni correction (*p* = 0.045). Similarly, parental depression symptoms at baseline were associated with lower Positive Parental Experience prior to Bonferroni adjustment (*p* = 0.024).

## 4 Discussion

This study investigated parental perceptions after 6 months of intensive therapist‐implemented Early Start Denver Model (ESDM) in 104 young autistic children. In line with our hypotheses, our findings extend previous research, showing that parents perceive meaningful benefits not only for their child, but also for themselves and their family.

### 4.1 Parental Perceptions of Child Progress

Parents reported improvements across all child domains. PCA identified three distinct factors: two related to the child (Social Communication, Nonverbal Social Engagement) and one related to the parents (Positive Parental Experience).

Regarding child‐related factors, the Social Communication factor explained the most variance in the PCA conducted on the Parental Perceptions of Change Questionnaire. The Social Communication factor aligns with core ESDM principles, which target social communication through imitation, play, and joint attention (Rogers 2016). This factor was strongly associated with the child's verbal and adaptive gains, supporting the validity of parental perceptions as meaningful outcome measures (McConachie et al. 2018) and suggesting that ESDM‐trained skills generalize beyond the intervention context. Additionally, this factor included the item on reduction of repetitive behaviors, reflecting the inverse association between social skills and repetitive and sensory‐motor behaviors reported in prior work (Chaxiong et al. 2022). Nevertheless, it is important to note that parents' familiarity with the objectives of ESDM, particularly the emphasis on social communication, may have influenced their responses toward these domains.

Moreover, the Nonverbal Social Engagement factor explained less variance in the PCA. While ESDM also targets nonverbal communication skills (e.g., eye contact, joint attention, facial expression) (Rogers 2016), these behaviors may emerge more slowly. For instance, gesture production increased after 1 year of intervention in a previous study (Logrieco et al. 2024). Nonverbal behaviors may also be less salient for parents than verbal communication, which tends to be a primary concern for parents of autistic children (Becerra‐Culqui et al. 2018; Kozlowski et al. 2011). Furthermore, social skills acquired in individualized sessions must generalize to peer interactions in natural contexts. Group‐based ESDM may better support such generalization by increasing opportunities for peer interactions (Feng et al. 2025; Vivanti et al. 2022).

Overall, these findings underscore the complementarity of subjective and objective perspectives. Parental reports of their child's development offer strong ecological validity by capturing changes in natural settings, while standardized assessments provide objective points of reference. Parent reports remain susceptible to bias (McConachie et al. 2018), and standardized assessments may underestimate abilities by measuring performance in a brief and unfamiliar context, where autistic children may have difficulty generalizing skills (Brown and Bebko 2012). Taken together, these findings support integrating both perspectives when evaluating early intervention outcomes.

### 4.2 Parental Perceptions of Their Experience

Parents reported reduced anxiety after 6 months of ESDM. This decrease may reflect the relief of accessing an intervention program and benefiting from coaching that provides guidance and emotional support. Engaging parents in intervention delivery has been shown to enhance parenting self‐efficacy and reduce stress (Bent et al. 2023; Estes et al. 2014; Pellecchia et al. 2023). A small subgroup, however, reported increased anxiety and perceived aggravations despite no objective regression over the six‐month period. This underscores the tight interplay between parental emotional state and perceptions of child progress, emphasizing the need to monitor parental mental health and to offer support ranging from informal parent networks to individualized support (e.g., mindfulness‐based therapy, cognitive behavior therapy, psychoeducation (Bent et al. 2023; Boonsuchat 2015; Da Paz and Wallander 2017; Neece et al. 2024; Ibrahim et al. 2025; Enav et al. 2019)). Alternatively, increased anxiety in these parents could reflect heightened awareness: as children begin early intervention, parents may more fully recognize their child's behaviors and difficulties, leading them to report ‘worsened’ skills that may indicate increased awareness rather than actual child regression.

Parents also reported gains in their own skills and in personal and familial well‐being, which were positively associated with perceived child progress across multiple domains (e.g., verbal communication, social reciprocity, broadening of interests). This pattern is consistent with a bidirectional parent–child dynamic, in which perceived child gains enhance parenting skills, which in turn may sustain parental engagement and use of intervention strategies at home (Karst and Van Hecke 2012; Enea and Rusu 2020).

However, the Positive Parental Experience factor did not correlate with standardized developmental measures, suggesting that despite modest child progress, support and coaching enhance parents' sense of control, positively influencing their experience regardless of measured outcomes. This represents an encouraging finding. It is also possible that the absence of correlation between Positive Parental Experience and developmental gains reflects the fact that changes in parental perceptions might require more time than the 6‐month intervention period to be fully detectable, and future research should examine longer‐term trajectories of parent experiences.

### 4.3 Parental Sociodemographic Characteristics and Their Implications

Our sample reflects considerable diversity in socioeconomic status, parental education, and languages spoken at home, consistent with the multicultural population of Geneva. This diversity is a strength, as it demonstrates the feasibility of engaging families across different backgrounds in early intervention programs, including those who may face barriers to participation (Malik‐Soni et al. 2022; Mendez et al. 2024). Importantly, this diversity contrasts with many previous studies of early intervention, which often include samples composed primarily of families with higher socioeconomic status and educational levels.

One likely explanation relates to the specific context of Geneva, Switzerland, where financial considerations do not constitute a major barrier to accessing diagnostic or early intervention services. In this context, inequalities in access to early diagnosis and intervention appear to be less pronounced than in many other countries. Diagnostic assessments are largely reimbursed through the health insurance system, and access to local early intervention programs does not depend on families' financial resources. This model likely facilitates access for families from a broader range of socioeconomic backgrounds and may explain the greater socioeconomic diversity observed in our sample. Taken together, these characteristics underscore the representativeness of our sample within the Geneva context and the value of examining early intervention outcomes in a diverse population.

Building on this description of parental and household characteristics, we examined how parental education related to parental experience in the ESDM program. Lower parental education was associated with more positive parental experiences, potentially reflecting greater perceived benefit from structured professional support. Conversely, highly educated parents may hold more demanding academic expectations or feel stronger pressure to maximize outcomes, which could moderate their perceived personal gains. This interpretation aligns with literature suggesting that parental expectations and stress are shaped by socioeconomic factors (Karst and Van Hecke 2012). Additionally, having more children in the household was linked to less positive parental experience, possibly due to increased caregiving demands, reduced time for the intervention, and challenges in balancing attention across children. Future studies should investigate how specific parental and familial sociodemographic features shape parental experience in ESDM.

### 4.4 Limitations and Methodological Reflections

The Parental Perceptions of Change Questionnaire was developed in 2014, shortly after the creation of the early intervention centers, as an initial effort to document parents' perceptions of a highly demanding intervention. Considering recent autism research, some items could be updated. For example, “Decrease of repetitive behaviors” is not a core ESDM target and these behaviors often increase in early childhood (Richler et al. 2010). Additionally, informed by the neurodiversity movement, aiming to reduce these behaviors raises ethical considerations, as they are recognized as part of autistic identity (Ne'eman et al. 2023).

Although the development of this questionnaire was informed by clinical experience and informal discussions with several parents, it did not involve a formal co‐creation process. Subsequent feedback from parent co‐authors during a dedicated session highlighted that parental well‐being is indeed a multidimensional construct and suggested that future instruments should better distinguish between emotional well‐being, social connectedness, professional balance, and leisure time. Parent co‐authors also stressed the importance of considering family context across important stages, from diagnosis to enrollment in early intervention, through to school entry. These insights highlight the value of involving parents from the outset through qualitative interviews or focus groups to support more participatory and co‐designed research approaches (Rabba et al. 2019; Boshoff et al. 2018; Bent et al. 2023; Edwards 2014).

Finally, the present study covered only the initial 6 months of ESDM. Future work should examine longer‐term perceptions, as well as the transition period that follows early intervention. Parents often report difficulties when moving away from early intervention services, where they previously received close attention, regular feedback, and structured guidance for preschool planning (Boshoff et al. 2018; Bent et al. 2023). Even when services are put in place to promote continuity of care and support parents in navigating educational systems beyond early intervention, transitioning to school often represents a period of intense and potentially stressful changes for children and families.

This study provides the first large‐scale examination of parental perceptions during intensive ESDM. Parents reported improvements in their child's development, their own skills, and family well‐being. These findings underscore parents' role as key stakeholders and outcome reporters. Given parents' central role in advocating, accessing early intervention, and implementing strategies at home, it is crucial to consider their impact within the parent–child transactional relationship. Future studies should combine qualitative and quantitative data to provide a richer understanding of intervention outcomes. Systematically addressing parental perceptions can enhance understanding of child and family outcomes, advancing a family‐centered model of early intervention that supports lasting developmental gains. Future work will also need to compare ESDM with other NDBIs or other interventions to investigate the specificity and generalizability of our findings.

## Funding

This work was supported by Schweizerischer Nationalfonds zur Förderung der Wissenschaftlichen Forschung, 190084, 202235, 212653. National Centre of Competence in Research (NCCR) Synapsy, 51NF40–185897. Fondation Privée des Hôpitaux Universitaires de Genève. Fondation Pôle Autisme.

## Ethics Statement

The Ethics Committee of the University of Geneva approved the research protocol.

## Consent

Written informed consent was obtained from the parents of all participating children.

## Conflicts of Interest

The authors declare no conflicts of interest.

## Supporting information


**Figure S1:** Change in parental anxiety and depression symptoms (HADS) from Baseline to Follow‐up (*N* = 91). Anxiety scores decreased significantly from Baseline (mea*n* = 7.32) to Follow‐up (mean = 6.54), *p* = 0.036, whereas depression scores remained stable (Baseline mean = 6.25; Follow‐up mean = 5.96), *p* = 0.888. The blue shaded area represents scores below the clinical threshold, with HADS scores ≥ 11 indicating clinical significance.
**Figure S2:** Radar chart showing responses of the five parents who reported slightly worsened, or greatly worsened skills in at least one child‐focused domain of the questionnaire. Each line represents the responses of one parent (Parent 1–5) across the 12 child‐focused question of the questionnaire. Responses were recoded: −3 (Greatly worsened), −2 (Worsened), −1 (Slightly worsened), 0 (Unchanged), 1 (Slightly improved), 2 (Improved), 3 (Greatly improved). The red area indicates negative perceptions.
**Figure S3:** Change in parental anxiety symptoms (HADS) from Baseline to Follow‐up. Comparison between parents who reported regression in at least one child‐focused domain over the last 6 months (*N* = 5) and parents who did not report any regression. One parent in the regression group did not complete the HADS, resulting in four data points instead of five. HADS, Hospital Anxiety and Depression Scale; Δ, HADS Anxiety Score at Follow‐up—HADS Anxiety Score at Baseline.
**Figure S4:** Scree plot of eigenvalues from Principal Component Analysis. The plot shows eigenvalues (*y*‐axis) for each component (*x*‐axis). An elbow is visible at the third component, supporting the retention of three factors for rotation and interpretation.
**Figure S5:** Spearman correlations between the 3 factors.
**Figure S6:** Significant Spearman correlations between Factor 1 (Social Communication) and the clinical variables.
**Figure S7:** Significant Spearman correlation between Factor 3 (Nonverbal Social Engagement) and VABS‐II Total Rate of Change.
**Figure S8:** Significant Spearman correlation between Factor 2 (Positive Parental Experience) and parental education, as well as the number of siblings.
**Table S1:** Distribution of responses to each questionnaire child‐focused item across response options, with column totals.
**Table S2:** Distribution of responses to each questionnaire child‐focused item across response options, with column totals.
**Table S3:** Wilcoxon signed‐rank test for the 18 items of the questionnaire. *N* represents the total number of responses for each item in the sample of 104 participants. *n* refers to the number of non‐zero responses included in the analysis.
**Table S4:** *p‐*values of the Spearman correlation matrix fro the 18 questionnaire items. Bold *p*‐values indicate survival after Bonferroni correction.
**Table S5:** Factor loadings of the 18 questionnaire items in the sample without missing data (*n* = 97).
**Table S6:** Spearman correlations between the 3 factors.

## Data Availability

The raw data supporting the conclusions of this article will be made available by the authors on a reasonable request.
