# Supplementary material for: Through Parents' Eyes: Perspectives on Child Development and Family Experience During ESDM Intervention
Source: Autism Res. 2026 Jul 25;19(9):e70318. doi: 10.1002/aur.70318 (PMC13599708; doi:10.1002/aur.70318)
Supplement: Supplementary file 1 [file AUR-19-0-s001.docx]

**Supplementary Information**

**Supplementary Figures**

**Figure S1.** Change in parental anxiety and depression symptoms (HADS) from Baseline to Follow-up (N = 91). Anxiety scores decreased significantly from Baseline (mean = 7.32) to Follow-up (mean = 6.54), p = 0.036, whereas depression scores remained stable (Baseline mean = 6.25; Follow-up mean = 5.96), p = 0.888. The blue shaded area represents scores below the clinical threshold, with HADS scores ≥11 indicating clinical significance.

**Figure S2.** Radar chart showing responses of the five parents who reported slightly worsened, or greatly worsened skills in at least one child-focused domain of the questionnaire. Each line represents the responses of one parent (Parent 1–5) across the 12 child-focused question of the questionnaire. Responses were recoded: -3 (Greatly worsened), -2 (Worsened), -1 (Slightly worsened), 0 (Unchanged), 1 (Slightly improved), 2 (Improved), 3 (Greatly improved). The red area indicates negative perceptions.

**Figure S3.** Change in parental anxiety symptoms (HADS) from Baseline to Follow-up. Comparison between parents who reported regression in at least one child-focused domain over the last six months (N = 5) and parents who did not report any regression. One parent in the regression group did not complete the HADS, resulting in four data points instead of five. HADS = Hospital Anxiety and Depression Scale; Δ = HADS Anxiety Score at Follow-up – HADS Anxiety Score at Baseline.

**Figure S4**. Scree plot of eigenvalues from Principal Component Analysis. The plot shows eigenvalues (y-axis) for each component (x-axis). An elbow is visible at the third component, supporting the retention of three factors for rotation and interpretation.

**Figure S5.** Spearman correlations between the 3 factors

**Figure S6.** Significant Spearman correlations between Factor 1 (Social Communication) and the clinical variables.

**Figure S7.** Significant Spearman correlation between Factor 3 (Nonverbal Social Engagement) and VABS-II Total Rate of Change.

**Figure S8.** Significant Spearman correlation between Factor 2 (Positive Parental Experience) and parental education, as well as the number of siblings.

**Supplementary Tables**

**Table S1.** Distribution of responses to each questionnaire child-focused item across response options, with column totals.

|  | Greatly worsened | Worsened | Slightly worsened | Unchanged | Slightly improved | Improved | Greatly improved | Total |
| --- | --- | --- | --- | --- | --- | --- | --- | --- |
| Time spent alone | 0 | 0 | 0 | 6 | 21 | 53 | 23 | 103 |
| Eye contact | 0 | 0 | 0 | 3 | 23 | 47 | 31 | 104 |
| Preferential relationships | 1 | 0 | 0 | 29 | 27 | 30 | 13 | 100 |
| Social reciprocity with children | 1 | 0 | 0 | 16 | 36 | 36 | 15 | 104 |
| Social reciprocity with parents | 0 | 0 | 0 | 5 | 21 | 52 | 26 | 104 |
| Play | 0 | 0 | 1 | 18 | 21 | 47 | 17 | 104 |
| Restricted interests | 0 | 0 | 1 | 4 | 26 | 48 | 25 | 104 |
| Repetitive behaviors | 1 | 0 | 2 | 16 | 33 | 38 | 13 | 103 |
| Non-verbal communication | 0 | 0 | 0 | 1 | 18 | 53 | 32 | 104 |
| Verbal communication | 0 | 0 | 0 | 6 | 28 | 32 | 38 | 104 |
| Autonomy | 0 | 0 | 0 | 4 | 24 | 52 | 24 | 104 |
| Attention | 0 | 0 | 1 | 2 | 25 | 49 | 27 | 104 |
| Eye contact | 0 | 0 | 0 | 3 | 23 | 47 | 31 | 104 |
| Time spent alone | 0 | 0 | 0 | 6 | 21 | 53 | 23 | 103 |

**Table S2.** Distribution of responses to each questionnaire child-focused item across response options, with column totals.

|  | Yes, enormously | Yes, a lot | Yes, a little | No | Total |
| --- | --- | --- | --- | --- | --- |
| Perceptions of people surrounding the family | 12 | 37 | 34 | 18 | 101 |
| Positive impact on family life | 24 | 31 | 21 | 8 | 104 |
| Positive impact on mood, work and social relationships | 27 | 39 | 22 | 6 | 104 |
| Handling tough times | 20 | 44 | 35 | 5 | 104 |
| Establishing rapport and communicating with your child | 34 | 51 | 18 | 1 | 104 |
| Understanding child needs | 37 | 45 | 21 | 1 | 104 |
| Positive impact on mood, work and social relationships | 27 | 39 | 22 | 6 | 104 |

**Table S3.** Wilcoxon signed-rank test for the 18 items of the questionnaire. ***N*** *represents the total number of responses for each item in the sample of 104 participants.* ***n*** *refers to the number of non-zero responses included in the analysis.*

|  | Wilcoxon Signed Rank Test | | | | | Effect size |
| --- | --- | --- | --- | --- | --- | --- |
|  | **W** | **N** | **n** | **Z** | ***p*** | ***r*** |
| Time spent alone | 4753 | 103 | 97 | 8.551 | <0.0001 | 0.868 |
| Eye contact | 5151 | 104 | 101 | 8.725 | <0.0001 | 0.868 |
| Preferential relationships | 2427 | 100 | 71 | 6.584 | <0.0001 | 0.781 |
| Social reciprocity with children | 3755 | 104 | 88 | 7.477 | <0.0001 | 0.797 |
| Social reciprocity with parents | 4950 | 104 | 99 | 8.638 | <0.0001 | 0.868 |
| Play | 3718 | 104 | 86 | 7.955 | <0.0001 | 0.858 |
| Restricted interests | 5022 | 104 | 100 | 8.585 | <0.0001 | 0.859 |
| Repetitive behaviors | 3595 | 103 | 87 | 7.115 | <0.0001 | 0.763 |
| Non-verbal communication | 5356 | 104 | 103 | 8.810 | <0.0001 | 0.868 |
| Verbal communication | 4851 | 104 | 98 | 8.595 | <0.0001 | 0.868 |
| Autonomy | 5050 | 104 | 100 | 8.682 | <0.0001 | 0.868 |
| Attention | 5226 | 104 | 102 | 8.678 | <0.0001 | 0.859 |
| Perceptions of people surrounding the family | 3486 | 101 | 83 | 7.913 | <0.0001 | 0.869 |
| Positive impact on family life | 4656 | 104 | 96 | 8.507 | <0.0001 | 0.868 |
| Positive impact on mood, work and social relationships | 4851 | 104 | 98 | 8.595 | <0.0001 | 0.868 |
| Handling tough times | 4950 | 104 | 99 | 8.638 | <0.0001 | 0.868 |
| Establishing rapport and communicating with your child | 5356 | 104 | 103 | 8.810 | <0.0001 | 0.868 |
| Understanding child needs | 5356 | 104 | 103 | 8.810 | <0.0001 | 0.868 |
| Time spent alone | 4753 | 103 | 97 | 8.551 | <0.0001 | 0.868 |

**Table S4.** *P-*values of the Spearman correlation matrix fro the 18 questionnaire items. Bold *p*-values indicate survival after Bonferroni correction.

|  | **Perceptions of people surrounding the family** | **Positive impact on family life** | **Positive impact on mood. work and social relationships** | **Handling tough times** | **Establishing rapport and communicating with your child** | **Understanding child needs** | **Attention** | **Autonomy** | **Non-verbal communication** | **Verbal communication** | **Reduction of repetitive behaviors** | **Broadening of interests** | **Play** | **Social reciprocity with parents** | **Social reciprocity with children** | **Eye contact** | **Preferential relationships** | **Time spent alone** |
| --- | --- | --- | --- | --- | --- | --- | --- | --- | --- | --- | --- | --- | --- | --- | --- | --- | --- | --- |
| **Perceptions of people surrounding the family** |  | **<0.001** | **<0.001** | **<0.001** | **<0.001** | **0.001** | 0.133 | 0.127 | 0.277 | **<0.001** | 0.046 | **0.001** | 0.172 | 0.003 | 0.061 | 0.063 | 0.257 | 0.045 |
| **Positive impact on family life** |  |  | **<0.001** | **<0.001** | **<0.001** | **<0.001** | **<0.001** | 0.135 | 0.185 | 0.188 | 0.095 | **<0.001** | 0.133 | 0.029 | 0.038 | **<0.001** | **0.002** | 0.003 |
| **Positive impact on mood. work and social relationships** |  |  |  | **<0.001** | **<0.001** | **<0.001** | 0.019 | 0.082 | 0.146 | 0.148 | 0.086 | **0.002** | 0.154 | **0.001** | 0.011 | **0.002** | 0.007 | **<0.001** |
| **Handling tough times** |  |  |  |  | **<0.001** | **<0.001** | **0.001** | **0.002** | 0.02 | 0.003 | **<0.001** | **<0.001** | 0.003 | **<0.001** | **0.001** | **<0.001** | **<0.001** | **<0.001** |
| **Establishing rapport and communicating with your child** |  |  |  |  |  | **<0.001** | **0.002** | **<0.001** | 0.005 | **<0.001** | **0.001** | **<0.001** | 0.017 | **<0.001** | **0.002** | **0.001** | **<0.001** | **<0.001** |
| **Understanding child needs** |  |  |  |  |  |  | **<0.001** | **<0.001** | **<0.001** | **<0.001** | **<0.001** | **<0.001** | **0.001** | **<0.001** | 0.043 | **<0.001** | 0.003 | **<0.001** |
| **Attention** |  |  |  |  |  |  |  | **<0.001** | 0.008 | **<0.001** | 0.004 | **<0.001** | **<0.001** | 0.004 | **<0.001** | **0.001** | **<0.001** | **<0.001** |
| **Autonomy** |  |  |  |  |  |  |  |  | **<0.001** | **<0.001** | **0.001** | 0.005 | **<0.001** | **<0.001** | **<0.001** | 0.005 | **<0.001** | **<0.001** |
| **Non-verbal communication** |  |  |  |  |  |  |  |  |  | 0.015 | **<0.001** | 0.099 | 0.008 | **<0.001** | **<0.001** | **<0.001** | **<0.001** | **<0.001** |
| **Verbal communication** |  |  |  |  |  |  |  |  |  |  | 0.038 | **<0.001** | **<0.001** | **<0.001** | **0.001** | 0.380 | 0.002 | **0.002** |
| **Reduction of repetitive behaviors** |  |  |  |  |  |  |  |  |  |  |  | **0.002** | 0.003 | 0.017 | 0.010 | **<0.001** | 0.004 | **0.001** |
| **Broadening of interests** |  |  |  |  |  |  |  |  |  |  |  |  | **<0.001** | **<0.001** | **0.001** | **<0.001** | **0.003** | **<0.001** |
| **Play** |  |  |  |  |  |  |  |  |  |  |  |  |  | **<0.001** | **<0.001** | **0.001** | **<0.001** | **<0.001** |
| **Social reciprocity with parents** |  |  |  |  |  |  |  |  |  |  |  |  |  |  | **<0.001** | **<0.001** | **<0.001** | **<0.001** |
| **Social reciprocity with children** |  |  |  |  |  |  |  |  |  |  |  |  |  |  |  | **<0.001** | **<0.001** | **<0.001** |
| **Eye contact** |  |  |  |  |  |  |  |  |  |  |  |  |  |  |  |  | **<0.001** | **<0.001** |
| **Preferential relationships** |  |  |  |  |  |  |  |  |  |  |  |  |  |  |  |  |  | **<0.001** |
| **Time spent alone** |  |  |  |  |  |  |  |  |  |  |  |  |  |  |  |  |  |  |

Principal component analysis

Principal component analysis (PCA) was performed on the 18 questionnaire items. Kaiser's criterion (eigenvalues > 1) suggested five components, but this method often overestimates factor numbers (Zwick & Velicer, 1986). The scree plot (Figure S4) showed a clearer break after the third component, supporting a three-factor solution. Thus, we re-ran PCA retaining three factors.

**Table S5**. Factor loadings of the 18 questionnaire items in the sample without missing data (n=97).

| **Questionnaire item** | **Factor 1 (38.35%)** | **Factor 2 (11.57%)** | **Factor 3 (8.07%)** |
| --- | --- | --- | --- |
| Verbal communication | **0.841** |  | -0.156 |
| Autonomy | **0.798** |  | 0.123 |
| Play | **0.596** | 0.125 | 0.263 |
| Social reciprocity with parents | **0.490** | -0.102 | 0.336 |
| Attention | **0.430** | -0.224 | 0.153 |
| Reduction of repetitive behaviors | **0.304** | -0.293 | 0.129 |
| Positive impact on mood, work and social relationships | -0.233 | **-0.846** | 0.119 |
| Positive impact on family life | -0.256 | **-0.843** | 0.168 |
| Handling tough times | 0.140 | **-0.706** | 0.129 |
| Perceptions of people surrounding the family | 0.125 | **-0.703** | -0.187 |
| Establishing rapport and communicating with your child | 0.284 | **-0.647** |  |
| Understanding child needs | 0.449 | **-0.554** |  |
| Broadening of interests | 0.316 | **-0.479** |  |
| Preferential relationships |  |  | **0.850** |
| Social reciprocity with children |  |  | **0.821** |
| Eye contact |  | -0.235 | **0.704** |
| Time spent alone | 0.143 | -0.143 | **0.640** |
| Non-verbal communication | 0.340 | 0.121 | **0.468** |
| Eigenvalues | 6.90 | 2.08 | 1.45 |
| % of variance | **38.35** | **11.57** | **8.07** |

**Table S6**. Spearman correlations between the 3 factors

|  | **Factor 1** | **Factor 2** | **Factor 3** |
| --- | --- | --- | --- |
| **Factor 1** | 1.00 | 0.266** | 0.443*** |
| **Factor 2** | 0.266** | 1.00 | 0.315** |
| **Factor 3** | 0.443*** | 0.315** | 1.00 |

Note: **p*<0.05, ***p*<0.01, ****p<*0.001
